# A Propagation Model of Social Hypernetwork Based on Directed Hypergraph

**DOI:** 10.3390/e28040420

**Published:** 2026-04-09

**Authors:** Lu Yang, Peng-Yue Li, Feng Hu, Zi-Ke Zhang

**Affiliations:** 1 School of Computer, Qinghai Normal University, Xining 810008, China; 2 The State Key Laboratory of Tibetan Intelligent Information Processing and Application, Xining 810008, China; 3 Center for Digital Communication Studies, Zhejiang University, Hangzhou 310058, China

**Keywords:** social hypernetwork, directed hypergraph, directed hypernetwork, information propagation

## Abstract

In the existing research on information propagation modeling in social networks, hypergraphs have been widely applied to characterize the high-order interaction relationships involving multiple nodes. However, most models are still based on the assumption of undirected connections, which leads to certain limitations in depicting the information flow direction and the structural characteristics of propagation chains. To address the above problems, a social hypernetwork propagation model with directional constraints is constructed in this paper by introducing the directed hypergraph structure and combining it with the improved SEIR model. The strength of social relationships is measured by intimacy in the model, and a comprehensive characterization of the information propagation process is achieved by integrating the threshold mechanism of the directed hypergraphs with the attenuation function of information timeliness. In addition, the effectiveness of the proposed model is verified by taking the event of “imposing additional tariffs” as an example, and the evolutionary characteristics of propagation in different network structures, as well as the impacts of user confidence and information timeliness, are analyzed using simulation experiments. The results indicate that the model is applicable to characterizing the information propagation trends and dynamic characteristics in real social networks, and can provide theoretical references and methodological support for the prediction and regulation of network public opinion.

## 1. Introduction

With the rapid development of internet technology, social media is regarded as the primary platform for information propagation, opinion expression, and public discussion [1,2,3], which exerts a profound influence on the evolution of public opinions and group behaviors. Information diffusion on such platforms generally presents directional characteristics [4] and is usually transmitted unidirectionally from partial nodes to other nodes along the paths of publishing, receiving and forwarding, thus forming a diffusion process with a hierarchical structure and constrained by propagation paths [5]. The systematic study on the directional structure in hypernetworks is of great significance for the construction of a theoretical framework that can accurately depict the actual propagation process of social media.

To depict the information diffusion process in social media more realistically, researchers have started to improve the network from the structural level. Traditional complex networks take binary edges as the basic connection units [6], and abundant achievements have been obtained in depicting node relationships and propagation structures. However, obvious limitations still exist in dealing with high-order propagation due to their inability to effectively express the synergy and group interaction behaviors among multiple nodes. To break through this limitation, the hypergraph model, which can connect multiple nodes simultaneously through a single hyperedge, has attracted extensive attention [7] and has gradually become an effective structural framework for depicting group behaviors, collective interactions, and high-order propagation dynamics. For example, Wang et al. [8] drew on the construction method of the WS small-world network, incorporated random hyperedge rewiring into the hypernetwork, and proposed a small-world hypernetwork model. Hu et al. [9] developed a hypernetwork evolution model based on the growth and preferential attachment mechanisms; theoretical analysis demonstrates that the node hyperdegree distribution of the model follows a power-law distribution, exhibiting scale-free characteristics. Xi et al. [10] proposed a weighted hypernetwork model (WKSN) that integrates two types of heterogeneous nodes—knowledge subjects and knowledge points—into a unified modeling framework. This model simulates the impact of brain drain on the organizational knowledge network through the associated node deletion method. Nevertheless, although the existing hypergraph models have structural advantages in depicting high-order interactions, most of them are based on the assumption of undirected connections [11,12,13,14], leading to certain limitations in describing the information flow direction and the structural characteristics of propagation chains. In contrast, information diffusion on actual social platforms usually has distinct directional characteristics: information diffuses from publishers to followers and is transmitted step by step along the forwarding links, and group influence also tends to present propagation characteristics directed from the influencing party to the influenced party [15]. These directional constraints all exert a profound influence on the propagation path, diffusion speed, and multi-level evolution process of information.

**Figure 1:**
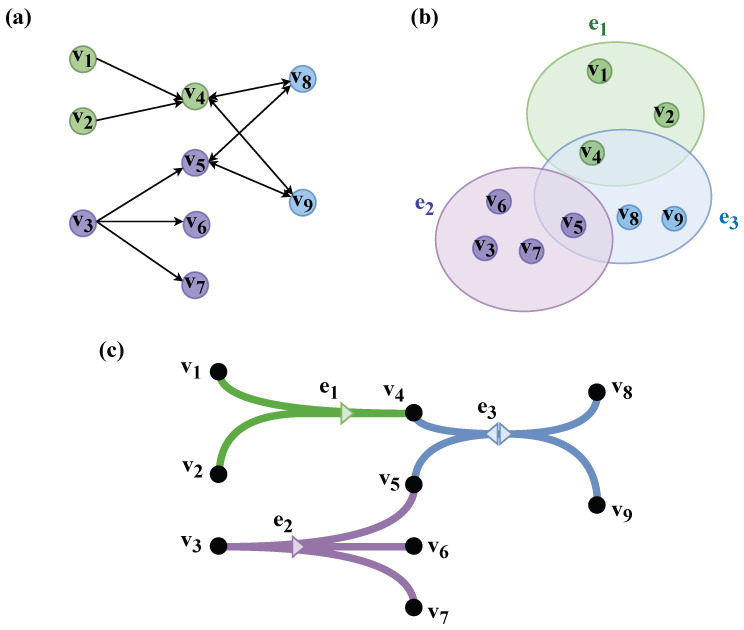
Illustration of different graph structures: (**a**) traditional directed graph, (**b**) hypergraph, and (**c**) directed hypergraph. These three structures are used to compare different modeling methods in social networks.

Undirected hypergraphs or simple binary directed edges cannot accurately depict the high-order interaction process with directional constraints. To break through this limitation, researchers have introduced directional descriptions into the hypergraph framework and constructed directed hypergraph structures that can distinguish the influencing party from the influenced party. Gallo G et al. [16] systematically defined the core concepts of directed hypergraphs. Cui et al. [17] constructed an SIS model with direct and indirect propagation paths for directed hypergraphs and proposed a competitive dual-virus model on directed hypergraphs. Burke Jamie et al. [18] introduced directed hypergraphs into the field of multimorbidity research, breaking the limitation that traditional directed graphs can only model pairwise relationships between diseases and realizing the modeling of high-order complex relationships in the temporal progression of multimorbidity. Xu et al. [19] proposed a configuration model for directed hypergraphs and studied the influence propagation of the threshold model on directed hypergraphs. Gallo et al. [20] proposed the M-directed hypergraph, a general framework capable of describing high-order directed interactions, and found that directed high-order interactions can bidirectionally regulate the stability of the synchronous state of nonlinear oscillators. Philip S. Chodrow et al. [21] proposed two types of random hypergraph configuration models, namely stub-labeled and vertex-labeled models, which can simultaneously retain the node degree sequence and edge dimension sequence. Kraakman et al. [22] proposed a Markov chain Monte Carlo sampling method based on double hyperedge shuffling, which can achieve uniform sampling of fixed vertex and hyperarc degree sequences in a specific directed hypergraph space. Studies from different perspectives have shown that directed hypergraphs provide a necessary structural basis for describing high-order propagation processes with directional constraints, thus demonstrating stronger descriptive capabilities in the research on information diffusion in real social networks. Figure 1 shows different modeling methods of follow relationships in actual social networks. Among them, Figure 1a represents the follow relationship in traditional complex networks, which can only depict the point-to-point connections between nodes; Figure 1b introduces the undirected hypergraph structure and incorporates group interactions into modeling, but cannot reflect the propagation direction of information along follow relationships due to the lack of directionality; Figure 1c adopts a directed hypergraph, which distinguishes the influencing party from the influenced party, making each hyperedge not only represent the high-order group relationship, but also clearly define the information propagation direction. This modeling method is more consistent with the information diffusion mechanism in actual social platforms, where information propagation usually has distinct directional characteristics, is transmitted from publishers to followers, and, at the same time, is highly dependent on the group interaction process with the joint participation of multiple users. For example, some authoritative nodes may be unidirectionally followed by a large number of users, while ordinary users may also form mutual follow relationships, thus making the network present a diversified and asymmetric interaction pattern.

Directed hypergraphs can simultaneously describe multi-node synergistic interactions and the directionality of information propagation within a unified framework, making them more suitable for characterizing the real information diffusion processes on social platforms. Therefore, this paper proposes a social hypernetwork propagation model based on directed hypergraphs, in which nodes represent users and directed hyperedges model social relationships, presenting the directional constraints and diversity of social relationships at the structural level. On this basis, the SEIR propagation mechanism is combined with the threshold model of directed hypergraphs to further enhance the ability to characterize the features of network information propagation. This model not only reveals the dynamic characteristics of information propagation in social media, but also provides a structured modeling foundation for the mechanism analysis of complex propagation processes and related applications. Section 1 of this paper is the Introduction, which elaborates on the research background of this study. Section 2 introduces the concepts of hypergraphs, directed hypergraphs, and directed hypernetworks. Section 3 focuses on the construction of the directed hypernetwork model, detailing its design principles and implementation process. Section 4 proposes an information propagation model in social hypernetworks, introduces the factors influencing propagation, and conducts an in-depth analysis of the propagation parameters. Section 5 carries out model validation and simulation analysis on the information propagation model in social hypernetworks, verifying the rationality and effectiveness of the model. Section 6 summarizes the main findings of this research and puts forward prospects for future research directions.

The main contributions of this paper are as follows:

(1) Aiming at the group interaction characteristics of the coexistence of one-way attention and mutual attention in social networks, a new directed hypernetwork information propagation model is proposed.
(2) Through a high-order structure, the model measures the strength of social relationships, integrates the directed hypergraph threshold mechanism, and reveals the group evolution law under the joint action of directed structure constraints and individual behaviors.
(3) Taking the directed hypernetwork as the underlying model, the influence of propagation parameters and topological structure on information propagation is simulated through simulation experiments, and the effectiveness of the proposed model is verified on different datasets.

## 2. Theoretical Background

### 2.1. Hypergraph

Let $V=\{v_{1},v_{2},\ldots ,v_{n}\}$ be a finite set [23]. If

(1) $e_{i}\neq Ø\;\left(i=1,2,\ldots ,m\right)$;
(2) ${⋃}_{i=1}^{m}e_{i}=V$.

then the binary relation H=(V,E) is called a hypergraph. The elements $v_{1},v_{2},\ldots ,v_{n}$ of *V* are referred to as the vertices of the hypergraph, and $E=\{e_{1},e_{2},\ldots ,e_{m}\}$ is the set of hyperedges of the hypergraph. Each set $e_{i}=\left\{v_{i1},v_{i2},\ldots ,v_{ij}\right\}$(i=1,2,…,m) is called a hyperedge of the hypergraph.

The hyperdegree of a node is defined as the number of hyperedges containing that node, denoted as $d_{H}\left(v_{i}\right)$. The hyperdegree distribution $P(d_{H})$ is defined as the ratio of the number of nodes $N_{d_{H}}$ with hyperdegree $d_{H}$ to the total number of nodes *N*, i.e.,

(1) $$P\left(d_{H}\right)=\frac{N_{d_{H}}}{N}$$

### 2.2. Directed Hypergraph

The definition of the directed hypergraph was systematically proposed in [16]. For an arbitrary unweighted directed hypergraph $\vec{G}=\left(V^{\prime },E^{\prime }\right)$, where $V^{\prime }=\left\{v_{1},v_{2},\ldots ,v_{n}\right\}$ denotes the vertex set in the directed hypergraph, and $E^{\prime }=\left\{e_{1},e_{2},\ldots ,e_{m}\right\}$ denotes the directed hyperedge set in the directed hypergraph. For any directed hyperedge *e* in the directed hypergraph, it can be expressed as $e=\{e_{\mathrm{tail}},e_{\mathrm{head}}\}$, where $e_{\mathrm{tail}}$ is the tail vertex set of the directed hyperedge and $e_{\mathrm{head}}$ is the head vertex set of the directed hyperedge, with $e_{\mathrm{tail}}\neq Ø$, $e_{\mathrm{head}}\neq Ø$ and $e_{\mathrm{tail}}\cap e_{\mathrm{head}}=Ø$.

In this paper, the incidence matrix $\vec{H}=\left\{{\vec{H}}_{\mathrm{tail}},{\vec{H}}_{\mathrm{head}}\right\}$ of the directed hypergraph is defined based on the combination of ${\vec{H}}_{\mathrm{tail}}$ and ${\vec{H}}_{\mathrm{head}}$, which represent the incidence of vertex *v* with the head $e_{\mathrm{head}}$ and tail $e_{\mathrm{tail}}$ of the directed hyperedge, respectively. They are expressed as follows in Equations (2) and (3):

(2) $${\vec{H}}_{\mathrm{head}}\left(v,e\right)=\left\{\begin{array}{cc} 1, & v\in e_{\mathrm{head}},\\ 0, & v\notin e_{\mathrm{head}}. \end{array}\right.$$

(3) $${\vec{H}}_{\mathrm{tail}}\left(v,e\right)=\left\{\begin{array}{cc} 1, & v\in e_{\mathrm{tail}},\\ 0, & v\notin e_{\mathrm{tail}}. \end{array}\right.$$

**Figure 2:**
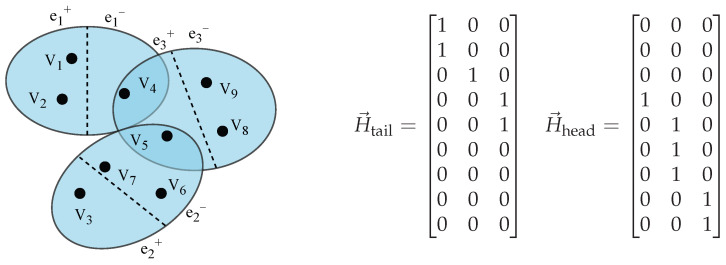
Structure and incidence matrix of the directed hypergraph. (**Left**) Schematic diagram of the directed hypergraph, where dashed lines are used to partition the internal structure of hyperedges to clarify the directional relationships between nodes, indicating that the influence propagates from the tail set $e^{+}$ to the head set $e^{-}$. Black dots represent nodes, and blue ellipses represent directed hyperedges. (**Right**) The corresponding incidence matrices ${\vec{H}}_{\mathrm{tail}}$ and ${\vec{H}}_{\mathrm{head}}$.

The structure of the directed hypergraph and its corresponding incidence matrix are shown in Figure 2. The directed hypergraph illustrated in the figure consists of nine vertices and three groups of directed hyperedges. For the convenience of subsequent representation, this paper adopts the method of set partitioning to represent the directed hypergraph. Each directed hyperedge is composed of two disjoint node subsets, namely $e_{\mathrm{tail}}$ and $e_{\mathrm{head}}$, which denote the tail node set and head node set of the hyperedge, respectively. In the figure, the internal structure of the hyperedge is divided by dashed lines to clarify the directional relationship between nodes, indicating that the action or influence is directed from the tail $e^{+}$ to the head $e^{-}$.

The degree of a vertex in a directed hypergraph is defined as the number of directed hyperedges in which the vertex participates, and the degree of a directed hyperedge is defined as the number of vertices contained in the hyperedge. The degrees of directed hyperedges and vertices can be calculated by using the incidence matrix, which are expressed as Equations (4) and (5), respectively [24]:

(4) $$\deg\left(e\right)=\sum_{v\in V}{\vec{H}}_{\mathrm{tail}}\left(v,e\right)+\sum_{v\in V}{\vec{H}}_{\mathrm{head}}\left(v,e\right)$$

(5) $$\deg\left(v\right)=\sum_{e\in E}{\vec{H}}_{\mathrm{tail}}\left(v,e\right)+\sum_{e\in E}{\vec{H}}_{\mathrm{head}}\left(v,e\right)$$

### 2.3. Threshold Model of Directed Hypergraph

To characterize the activation behavior of nodes in a directed hypergraph, the node state is defined by adopting a threshold update rule based on the number of activated in-neighbors [19], as follows:

(6) $$X_{i}\left(0\right)=\sigma_{i},$$

The state evolves according to

(7) $$X_{i}\left(t+1\right)=\left\{\begin{array}{cc} 1, & m_{i}\left(t\right)\geq \theta_{i}K_{i},\\ 0, & m_{i}\left(t\right)<\theta_{i}K_{i}. \end{array}\right.$$

where t≥0 and i=1,…,n. $K_{i}$ denote the number of in-neighbors of node $v_{i}$, referring to the total number of nodes that can directly affect the target node $v_{i}$ via directed hyperedges. $m_{i}\left(t\right)$ denotes the number of activated in-neighbors of node $v_{i}$, and $\theta_{i}$ is the activation threshold of node $v_{i}$.

### 2.4. Directed Hyperwork

**Figure 3:**
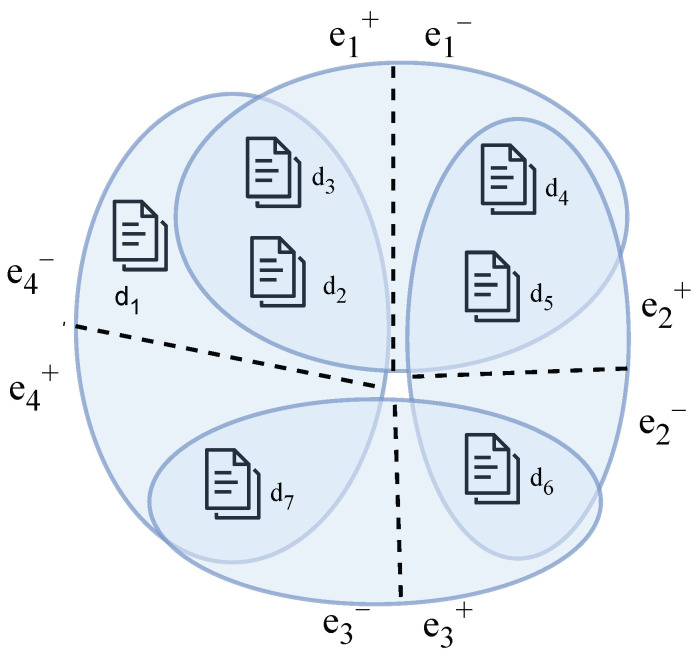
Schematic diagram of the directed hypernetwork for the citation network, where blue ellipses represent directed hyperedges, describing one-to-one, one-to-many, many-to-one and many-to-many citation relationships.

Directed hypernetwork is an important network model for characterizing asymmetric high-order interaction relationships in complex systems. Its structural foundation is derived from hypergraph theory [25], and the synergy among multiple nodes is uniformly described by hyperedges, thus breaking through the limitations of traditional binary relational networks in terms of high-order interactions. A hypernetwork is defined as an undirected hypernetwork when the hyperedges in the hypernetwork do not distinguish the direction of action and the order of priority, and the interactions between nodes are regarded as symmetric relationships. In contrast, it is defined as a directed hypernetwork when the hyperedges clearly distinguish the influencing parties from the influenced parties, and information or actions propagate from one set of nodes to another set of nodes. By retaining the high-order interaction structure and introducing directional constraints simultaneously, the directed hypernetwork provides a more realistic network description framework for characterizing complex systems with asymmetric characteristics. Figure 3 presents a schematic diagram of the directed hypernetwork for citation relationships among articles, where nodes represent articles and directed hyperedges represent citation relationships, with the citation direction pointing from the citing papers $e^{+}$ to the cited papers $e^{-}$.

## 3. Directed Hypernetwork Model Construction

**Figure 4:**
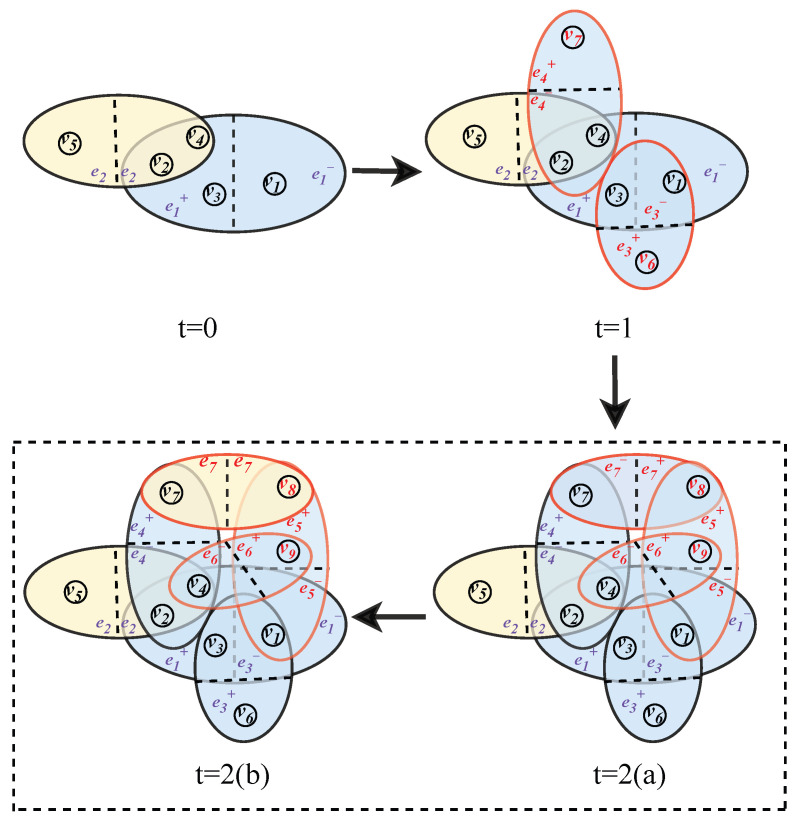
Schematic diagram of the directed hypernetwork evolution process, where nodes represent users and hyperedges represent follow relationships.

Based on the BA scale-free hypernetwork model [9], this paper introduces directionality to construct a directed hypernetwork model, which is adopted as the underlying topological structure of the proposed model. The construction process is as follows:

(1) **Initialization**: The initial directed social hypernetwork contains $n_{0}$ users, and the initial group relationships are established through two types of hyperedges: one-way follow and mutual follow.
(2) **Growth**: At each time step *t*, $n_{1}$ new nodes are added to the network and establish directed connections with $n_{2}$ existing nodes in the current network, which simulates the process of users continuously joining the network and forming social connections. Two types of directed hyperedges are formed during the node addition process: if a new node follows an existing node in the network, a one-way follow hyperedge is constructed; if the existing node follows the new node back within the same time step, the original one-way follow hyperedge is converted into a mutual follow hyperedge.
(3) **Preferential Attachment**: The probability that a newly added node establishes connections with existing nodes is jointly determined by the in-degree and out-degree of the target node. The in-degree characterizes the attention degree of the node, representing the number of followers of the node. The out-degree reflects the social activity of the node, representing the number of followings of the node. The probability of connecting to the target node is defined as Figure 4 illustrates the evolution process of the directed hypernetwork, where the blue ellipses represent one-way follow hyperedges and the yellow ellipses represent mutual follow hyperedges. Nodes on both sides of the dashed line form follow relationships with each other, while no follow relationship exists between nodes on the same side of the dashed line. The direction of hyperedges is indicated by blue marks, and the newly added nodes and hyperedges at each time step are marked in red. At t=0, nodes $v_{2}$, $v_{3}$ and $v_{4}$ jointly follow node $v_{1}$ in a one-way manner, forming a one-way follow hyperedge $e_{1}$ with the head set $\left\{v_{1}\right\}$ and the tail set $\left\{v_{2},v_{3},v_{4}\right\}$; nodes $v_{2}$ and $v_{5}$ follow each other mutually, forming a mutual follow hyperedge $e_{2}$. At t=1, new nodes $v_{6}$ and $v_{7}$ are added to the network: node $v_{6}$ follows nodes $v_{1}$ and $v_{3}$ in a one-way manner, forming a one-way follow hyperedge $e_{3}$; node $v_{7}$ follows nodes $v_{2}$ and $v_{4}$ in a one-way manner, forming a one-way follow hyperedge $e_{4}$. At t=2, new nodes $v_{8}$ and $v_{9}$ are added to the network, and the evolution at this time step is further divided into two phases. In phase t=2(a), nodes $v_{8}$ and $v_{9}$ jointly follow node $v_{1}$ in a one-way manner, forming a one-way follow hyperedge $e_{5}$; nodes $v_{8}$ and $v_{9}$ also follow nodes $v_{7}$ and $v_{4}$, respectively, in a one-way manner, forming two one-way follow hyperedges $e_{7}$ and $e_{6}$, where hyperedge $e_{6}$ has the head set $\left\{v_{4}\right\}$ and the tail set $\left\{v_{9}\right\}$, and hyperedge $e_{7}$ has the head set $\left\{v_{7}\right\}$ and the tail set $\left\{v_{8}\right\}$. In phase t=2(b), node $v_{7}$ follows node $v_{8}$ back, and the two nodes jointly form a mutual follow hyperedge, which indicates that the hyperedge is transformed from a one-way follow relationship to a mutual follow relationship.

## 4. Information Propagation Model on Social Hypernetworks

Information propagation on social platforms is jointly influenced by the synergistic effects of group interaction structures and propagation directionality constraints, exhibiting complex and diverse dynamic evolution characteristics. To elaborate on the propagation process under such high-order interactions, a social hypernetwork propagation model based on directed hypergraphs is constructed in this section. By systematically characterizing the node state evolution, propagation directionality constraints, and the action mechanisms of key propagation parameters, the proposed model can effectively analyze the diffusion behavior and dynamic evolution laws of information in social hypernetworks, thus providing a clear modeling foundation for subsequent simulation experiments and empirical analyses.

### 4.1. SEIR Information Propagation Process on Directed Hypernetworks

In the process of information propagation on social hypernetworks, network users are divided into the following four states:

- **Susceptible (S)**: Nodes that have not obtained the information and are unaware of it.
- **Exposed (E)**: Nodes that are aware of the information but do not propagate it.
- **Infected (I)**: Nodes that are aware of the information and are propagating it actively.
- **Recovered (R)**: Nodes that are aware of the information, but have no interest in continuing to propagate it.

**Figure 5:**
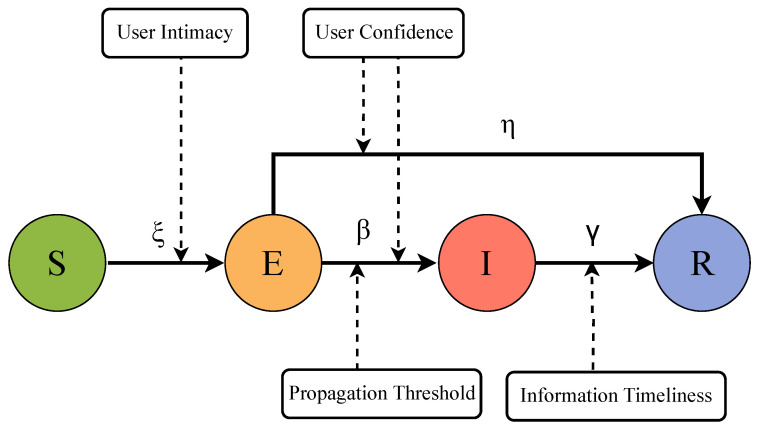
Diagram of information propagation process.

The information propagation process on the social hypernetwork based on directed hypergraphs is illustrated in Figure 5, and the detailed description is as follows:

(1) **Initialization**: All nodes in the hypernetwork are set as susceptible nodes (S-state) that have not received any information.
(2) **Source Node Selection**: Nodes accounting for a proportion of $\rho_{0}$ are randomly selected as infectious nodes (I-state), and the remaining nodes are all susceptible nodes (S-state).
(3) **Information Propagation**: During the information propagation process, nodes in the I-state propagate information to S-state nodes along the direction of directed hyperedges. The S-state nodes receiving the information judge the credibility of the information based on the intimacy value with the I-state nodes and accept the information with a probability of ξ, thus transforming from the S-state to the E-state. The transformation of E-state nodes to I-state nodes depends on the propagation threshold of nodes and the state of their in-neighbors. The propagation threshold of each node is positively correlated with its ability to judge information, namely, the user confidence. When the condition of the minimum number of active influence sources is satisfied, the node transforms from the E-state to the I-state with a probability of β and starts to propagate information or transforms to the R-state with a probability of η. Information propagation modes vary among different types of hyperedges: for mutual follow hyperedges, information can be propagated mutually between the two connected nodes; for one-way follow hyperedges, the information propagation direction is opposite to the follow direction, namely, information is propagated from the followed nodes to the following nodes. As time elapses, I-state nodes lose their interest in propagation with a probability of γ and transform to the R-state; R-state nodes no longer transform to any other states and exit the propagation process.
(4) **Steady State**: With the continuous propagation of information in the hypernetwork, nodes in different states will reach a relatively stable state, which means that the information propagation in the hypernetwork achieves a steady state.

Assume that at time *t*, the numbers of users in the Susceptible (S(t)), Exposed (E(t)), Infected (I(t)), and Recovered (R(t)) states are continuous and differentiable. According to the above propagation rules, the change in the number of susceptible nodes per unit time is mainly caused by their receiving information and transforming into exposed nodes. The change in the number of exposed nodes increases, on the one hand, due to susceptible nodes receiving information, and decreases, on the other hand, due to some exposed nodes entering the infectious state or losing interest in the information and transforming into the recovered state. The change in the number of infectious nodes increases due to exposed nodes transforming into infectious nodes and decreases due to infectious nodes losing propagation interest and transforming into the recovered state. The change in the number of recovered nodes increases due to exposed and infectious nodes becoming immune to the information. The dynamic evolution equations and parameter constraints of the model are given by

(8) $$\left\{\begin{array}{c} \frac{dS(t)}{dt}=-S\left(t\right)I\left(t\right)\xi ,\\ \frac{dE(t)}{dt}=S\left(t\right)I\left(t\right)\xi -\left(\beta +\eta \right)E\left(t\right),\\ \frac{dI(t)}{dt}=\beta E\left(t\right)-\gamma I\left(t\right),\\ \frac{dR(t)}{dt}=\eta E\left(t\right)+\gamma I\left(t\right). \end{array}\right.$$

(9) $$\left\{\begin{array}{c} S(t)+E(t)+I(t)+R(t)=1,\\ \xi >0,\;\beta >0,\;\eta >0,\;\gamma >0. \end{array}\right.$$

**Figure 6:**
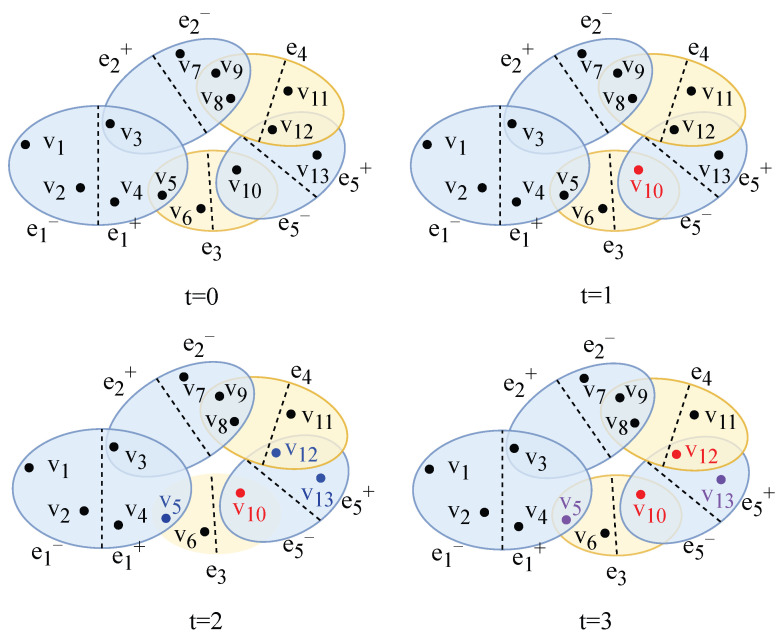
Schematic diagram of the propagation process in directed hypernetworks, where nodes represent users, blue ovals represent unidirectional follow hyperedges, and yellow ovals represent bidirectional follow hyperedges.

The four sub-equations in Equation (8) represent the change rates of the number of users in different states with time, and the sub-equations in Equation (9) represent the parameter constraint conditions in the propagation model. Figure 6 shows the schematic diagram of information propagation in the directed hypernetwork.

Figure 6 shows the propagation schematic of directed hypernetworks. Node colors represent different states: black denotes the susceptible state (S), blue denotes the exposed state (E), red denotes the infected state (I), and purple denotes the recovered state (R). At t=0, all nodes in the network are in the S state. At t=1, ρ=0.1 of nodes are randomly selected from the network (node $v_{10}$ in the schematic) as the initial infection sources, which are transferred into the infected state and start to propagate information outward. Then at t=2, since hyperedge $e_{3}$ is a mutual hyperedge where bidirectional propagation occurs among its nodes, node $v_{5}$ receives information propagated from node $v_{10}$ and remains in the E state temporarily without further propagation, while node $v_{6}$ does not receive information and is still in the S state. Hyperedge $e_{5}$ is a unidirectional following hyperedge, in which nodes in the tail set point to nodes in the head set, and information propagates along the reverse direction of following in the hypernetwork. Therefore, nodes $v_{12}$ and $v_{13}$ receive information propagated from node $v_{10}$ in the head set and are transferred into the E state. At t=3, node $v_{5}$ cannot continue to propagate due to the absence of out-neighbors and is transferred into the R state because of interest attenuation. The number of activated in-neighbors of node $v_{12}$ exceeds its minimum number of active influence sources, so it is transferred from the E state to the I state and starts to propagate information. Node $v_{13}$ does not reach the threshold and its interest weakens, so it is transferred into the R state with probability η. This process demonstrates the dynamic characteristics of the threshold-based SEIR model under the directed hypergraph structure in terms of propagation paths, state evolution and interest attenuation mechanism.

### 4.2. Influencing Factors of Information Propagation

In social networks, users usually acquire and update information from the objects they follow, and the information receiving and subsequent propagation processes are dynamically influenced by factors such as the structural characteristics of users’ social relationship networks, as well as individuals’ cognitive and judgment abilities. Based on this, this paper constructs an underlying directed hypernetwork and conducts quantitative modeling of the key propagation influencing factors to analyze the information propagation process and its evolution laws in the directed hypernetwork.

#### 4.2.1. Node Intimacy

To characterize the hierarchical differences in the strength of user relationships in social networks, node intimacy is introduced as a quantitative index in this paper, aiming to depict the intensity of trust and interaction between nodes. The intimate relationship between two adjacent individuals *i* and *j* is defined as the number of common neighbors, denoted as $n_{ij}$. In the directed hypergraph structure, the influence set of a node consists of all its in-neighbors, namely, the set of all tail nodes pointing to the node through directed hyperedges. The “common neighbors” of two nodes refer to the intersection of their influence sets, namely, the common in-neighbors pointing to both nodes simultaneously. Based on this, the intimacy between node *i* and node *j* is defined as follows [26]:

(10) $$\omega_{ij}=\frac{{\left(n_{ij}+1\right)}^{\alpha }}{E\left[{\left(n_{ij}+1\right)}^{\alpha }\right]},$$

where the constant 1 is introduced to avoid the meaninglessness of the formula caused by a zero denominator. The denominator term represents the average value of the number of common neighbors for all edges to achieve standardization. The parameter α serves as a weight factor for the intimacy between nodes, which is used to adjust the influence degree of node intimacy on hyperedge weights: when α>1, stronger node intimacy has a more significant impact on hyperedge weights, illustrated by closely connected small groups in social networks; when α<1, relatively weakened intimacy relationships are enhanced; when α=1, the formula is equivalent to the ordinary average method, and the weights are calculated in an unbiased manner.

#### 4.2.2. User Confidence

User confidence refers to the ability of users to judge the authenticity, reliability of received information, and the credibility of its source, which is an important factor affecting information adoption and re-propagation behaviors in social networks. In the social media environment, the information volume is very large and the quality varies significantly, and users’ judgment on information credibility largely determines whether they accept the information and continue to propagate it. Higher user confidence helps to improve the effectiveness of information screening, making information propagation more dependent on content quality and source reliability; on the contrary, lower user confidence may weaken users’ discrimination ability and increase the risk of unreliable information being adopted and diffused. The formula for user confidence is defined in [27] as

(11) $$C\left(v_{i}\right)=1-e^{-w(v_{i})},$$

where $w(v_{i})$ is a constant reflecting the individual’s ability to judge information, and its value level affects the overall trust tendency of users. To characterize individual differences among users, $w(v_{i})$ is set to obey a normal distribution:

(12) $$w\left(v_{i}\right)∼N\left(coi,\sigma_{coi}^{2}\right),$$

where coi denotes the mean value of $w(v_{i})$ and $\sigma_{coi}^{2}$ denotes the standard deviation. Attributes such as users’ educational level and cultural diversity will affect their information judgment ability. A higher value of $w(v_{i})$ leads to a higher value of the corresponding $C(v_{i})$, thus increasing users’ trust in information.

#### 4.2.3. Information Timeliness

Information timeliness refers to the characteristic that the relevance and value of information content change with time within its effective propagation cycle. In the social media environment, the speed of information update is extremely fast, and users’ focus has significant time sensitivity. Therefore, the propagation ability of information usually decays rapidly as time elapses after its release. Information with high timeliness, such as hot events and breaking news, has strong attractiveness and discussibility in a short time, and is more likely to be adopted and widely diffused by users; meanwhile, information with low timeliness has a corresponding decrease in propagation probability and influence range due to the reduction of its relevance with time. Reference [28] assumes that the immune rate at which information propagators gradually withdraw from the information propagation process changes exponentially with time, which is defined as

(13) $$1-e^{-\lambda t},$$

where λ is the timeliness characteristic of the network and *t* is the time step of the current information.

### 4.3. Propagation Parameters

State transition probabilities are important parameters for the changes in user states during the network information propagation process. The differences in influencing factors such as user intimacy, user confidence, and information timeliness in online social hypernetworks lead to the complexity of user state changes. Therefore, the state transition probabilities are defined as follows:

ξ: The probability that a node transforms from the S-state to the E-state, which is related to the intimacy between two nodes, defined as

(14) $$\xi \left(i,j\right)=\left\{\begin{array}{cc} \omega_{ij}, & \omega_{ij}\leq 1\\ 1, & \mathrm{otherwise} \end{array}\right.$$

β: The probability that a node transforms from the E-state to the I-state, which is related to the number of in-neighbors of the node, the number of activated in-neighbors of the node, and user confidence, defined as

(15) $$\beta \left(i,j\right)=\left\{\begin{array}{cc} 1, & \mathrm{if}\;m_{i}\left(t\right)\geq Cfds\cdot K_{i}\\ 0, & \mathrm{if}\;m_{i}\left(t\right)<Cfds\cdot K_{i} \end{array}\right.$$

η: The probability that a node transforms from the E-state to the R-state, which is related to the node’s ability to judge information (i.e., user confidence), defined as

(16) $$\eta \left(i,j\right)=\left\{\begin{array}{cc} C(v_{i}), & C(v_{i})\leq 1\\ 1, & \mathrm{otherwise} \end{array}\right.$$

γ: The probability that a node transforms from the I-state to the R-state, which is assumed to be only related to information timeliness, defined as

(17) $$\gamma \left(t+1\right)=1-e^{-\lambda t}$$

## 5. Model Validation and Simulation

In this section, a directed hypernetwork model is constructed via computer simulation, and the simulation results are compared with actual data to verify the effectiveness of the proposed model. On this basis, a series of simulation experiments were carried out to systematically analyze the impacts of different hyperdegree distributions, network structural characteristics, threshold settings, user confidence, information timeliness, and comparisons with the basic hypergraph model on the dynamic and evolutionary laws of information propagation. To reduce the deviation caused by randomness, each experiment was independently repeated 50 times, and the average value of the final results was adopted. Error bars corresponding to the 50 repeated experiments are plotted in the experimental figures to reflect the fluctuation range and stability of the results.

### 5.1. Hyperdegree Distribution of the Directed Hypernetwork

To verify the hyperdegree distribution characteristics of the network generated by the proposed model, a directed social hypernetwork with the parameters N=5000, $n_{0}=6$, $n_{1}=2$, $n_{2}=5$ and ε=0.8 was constructed. The effectiveness of the model in depicting high-order structural characteristics was evaluated through statistical analysis of the hyperdegree distribution.

**Figure 7:**
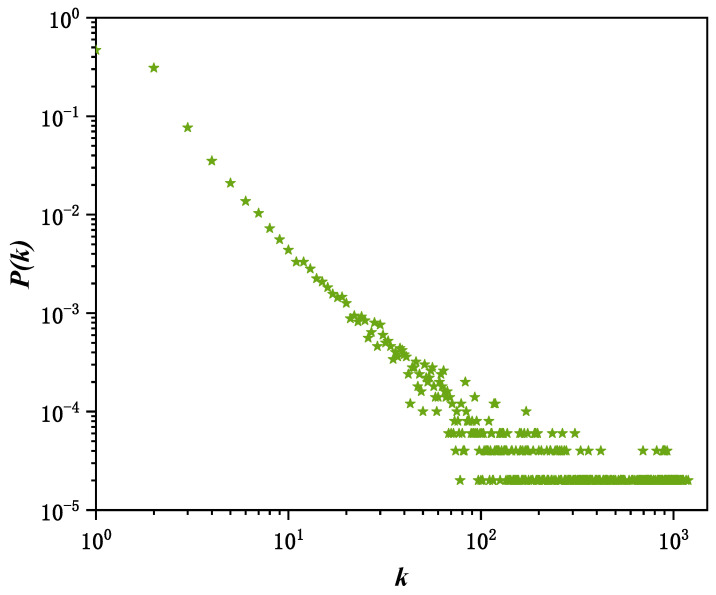
Simulating the hyperdegree distribution of directed social hypernetworks. The green stars in the figure represent data points, with each star corresponding to an observed transcendence value k and its corresponding probability P(k).

Figure 7 shows the distribution of hyperdegrees in the directed hypernetwork under double-logarithmic coordinates, where the horizontal axis represents the node hyperdegree and the vertical axis represents the corresponding degree distribution. It can be observed that the node hyperdegree distribution follows a power-law form, indicating that the constructed social network has scale-free characteristics: a small number of nodes participate in a large number of high-order interactions, while most nodes only involve a small number of hyperedge connections. This structural characteristic is consistent with the research conclusions on hyperdegree distribution in existing social hypernetwork and evolutionary hypergraph models [29], and also matches the statistical characteristics of user participation behaviors on real social media platforms [30]. A small number of head users or core accounts often participate frequently in multi-user interactions, topic discussions, or group propagation events, playing a key role in the information diffusion process. In contrast, most ordinary users have relatively limited participation, mainly acting as information receivers, thus forming an obvious “long-tail effect”. The results show that the directed hypernetwork model can well characterize the distribution characteristics of user participation in high-order interactions on social platforms.

### 5.2. Model Validation

#### 5.2.1. Model Validation on Public Datasets

To verify the applicability of the proposed model at the topological level, nine real-world public network datasets from five different fields are selected for comparative analysis [31]. Specifically, these datasets include the following:

(1) Metabolic networks (iAF1260b and iJO1366), in which nodes represent genes and hyperedges represent chemical reactions among different genes;
(2) Citation networks (DBLP-data-mining and DBLP-software (https://dblp.org/)), in which nodes represent researchers, and the head set and tail set of each hyperedge jointly indicate the citation relationship in a paper;
(3) Q&A networks (MathOverflow and StackExchange Server Fault), in which nodes represent users, each hyperedge corresponds to a post, with the questioner forming one end of the hyperedge and the respondents forming the other end;
(4) Bitcoin transaction networks (Bitcoin-2014, Bitcoin-2015 and Bitcoin-2016), in which nodes represent accounts and each hyperedge denotes a transaction between users.

The dataset topology is shown in Table 1.

This study selects nine real-world public network datasets covering fields such as question answering, metabolism, citation, and Bitcoin transactions. By comparing their topological statistical characteristics (node hyperdegree and power-law exponent γ) with those of networks generated by the proposed model, we find that, although the datasets differ significantly in scale, their topological structures exhibit consistent common regularities. Specifically, the minimum in-degree and minimum out-degree of all networks are both 1, indicating that no isolated nodes exist in these networks. The significant imbalance between in-degree and out-degree effectively characterizes the asymmetric differences in the directionality of network connections. Meanwhile, the power-law fitting exponents of in-degree, out-degree, and total hyperdegree mostly fall within the range of 1.77–2.40, suggesting that these real networks generally present heavy-tailed distributions. In comparison, the directed social hypernetwork generated by our model is consistent with the real datasets in these key topological properties. This demonstrates that the proposed model is not limited to capturing the structural characteristics of a specific sample, but can more generally reproduce the common topological properties observed in real-world directed hypergraphs, thus verifying its applicability at the topological level.

#### 5.2.2. Validation of the Social Network Model

**Figure 8:**
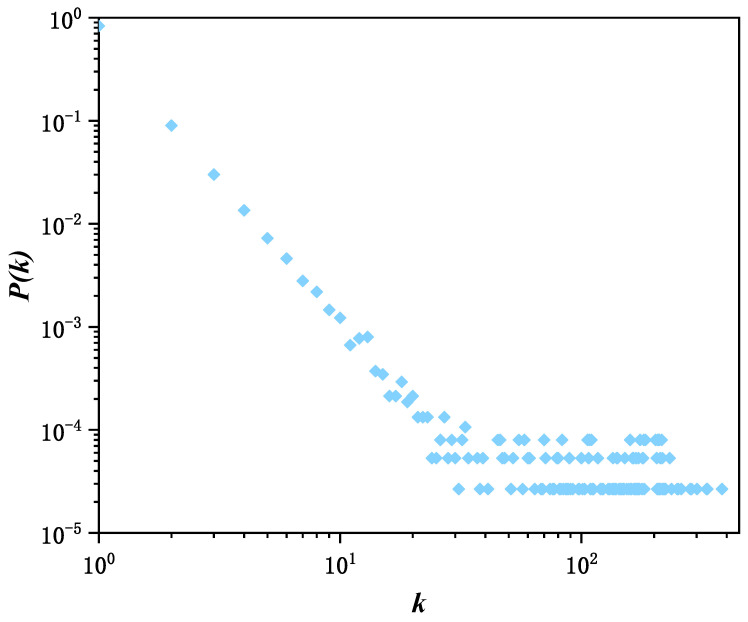
Hyperdegree distribution of empirical data. The blue diamonds in the figure are data points, each corresponding to an observed degree value k and its corresponding probability P(k).

**Figure 9:**
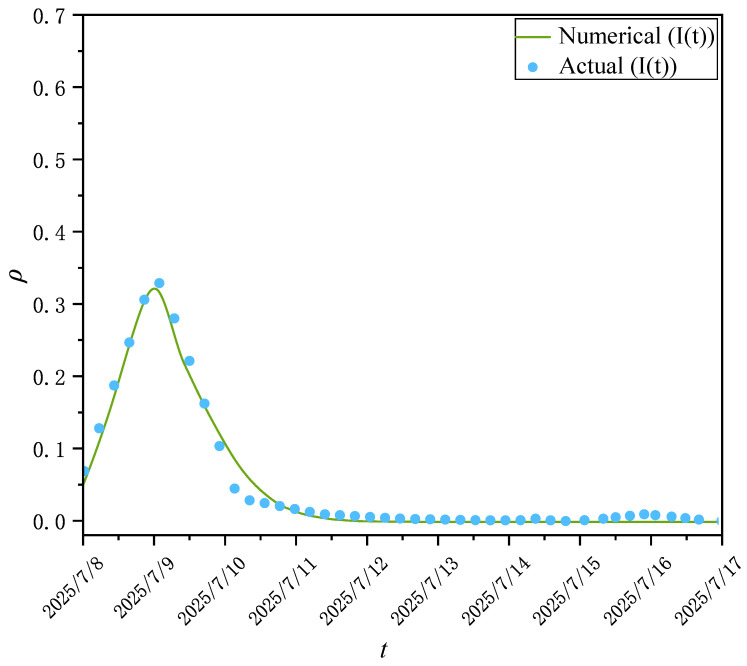
Comparison between the volume of user discussions and the number of nodes in I-state of the simulated directed social hypernetwork propagation model.

Since 2025, the economic and trade game between China and the United States around reciprocal tariffs has triggered sustained and extensive public discussion on social media. As an important economic regulation tool, tariff adjustments affects prices, employment, enterprise costs, and consumer welfare, and easily stimulates social emotions and value judgments, making the social communication of relevant issues present characteristics such as large scale, numerous participants, strong divergence of viewpoints, and fluctuating and decaying heat with the negotiation process. To systematically analyze the information transmission dynamics and group emotion evolution paths in complex economic events, this paper selects the “tariff imposition” event to carry out an empirical study. Based on the Sina Weibo platform, with “tariff imposition”, “Sino-US trade war”, and other keywords as the core, 397 relevant topics during the event fermentation period from 8 July 2025 to 17 July 2025 are retrieved. The topic discussion volume is taken as the core communication intensity indicator, and information such as user ID, release time, interaction behavior, user follower count and following count under the topics is collected simultaneously. After data cleaning, deduplication, time alignment, and user screening, valid interaction records were retained, on which a directed social hypernetwork was constructed. Meanwhile, simulation experiments were carried out on the directed hypernetwork with parameters N=5000, $n_{0}=6$, $n_{1}=2$, $n_{2}=5$ and ε=0.8. Figure 8 shows the hyperdegree distribution of user follow relationships on the Weibo platform under double-logarithmic coordinates. Figure 9 shows the comparison curves between the user discussion volume and the distribution of nodes in I-state of the simulated directed social hypernetwork propagation model.

Figure 8 shows the distribution of node hyperdegrees in the directed network constructed based on Weibo follow relationships under double-logarithmic coordinates, where the horizontal axis represents the hyperdegree and the vertical axis represents the corresponding degree distribution. It can be observed that the node hyperdegree distribution presents a long-tail characteristic and approximately follows a power-law distribution under double-logarithmic coordinates, which is highly consistent with the simulation results in Figure 7. This indicates that the directed social hypernetwork constructed in this paper conforms to scale-free characteristics and can be well used as the underlying network structure to depict the information propagation process.

Figure 9 compares the actual observed data of user discussion volume varying with time and the simulation curve of infected node density I(t) in the directed social hypernetwork propagation model. The horizontal axis denotes the event discussion date, the vertical axis denotes the corresponding topic discussion density, the blue scatter points represent the actual observed values of discussion volume on social platforms, and the green solid line denotes the variation trend of propagation node density simulated by the model. The results show that the two curves have a high degree of consistency in the overall shape, both presenting typical public opinion propagation characteristics: at the initial stage of the event outbreak, the discussion popularity rises rapidly and reaches a peak in a short time; then it gradually attenuates and finally tends to be stable and close to zero. The model achieves a good fit to the actual data, which indicates that the propagation mechanism described by the directed hypernetwork propagation model can effectively reflect the diffusion process of event information and the dynamic evolution law of public discussions on social platforms, thus verifying the effectiveness of the proposed model in real public opinion propagation scenarios.

### 5.3. Simulation Analysis of Different Hypernetwork Structures

**Figure 10:**
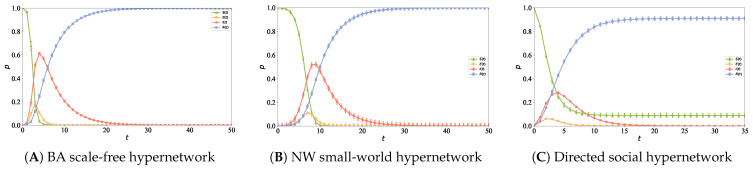
The information propagation curves differ significantly across various network structures, with (**A**) corresponding to the BA scale-free hypernetwork, (**B**) to the NW small-world hypernetwork, and (**C**) to the directed social hypernetwork, highlighting the structural effects on information spread in hypernetworks.

To investigate the process and laws of information propagation in directed hypernetworks, three different network structures are constructed in this paper, and the node with the maximum hyperdegree in the network is selected as the initial informed node. First, a BA scale-free hypernetwork with N=5000, $n_{0}=6$ and $n_{2}=5$ is generated, with a maximum hyperdegree of 343. Second, an NW small-world hypernetwork with N=5000, K=14 and rewiring probability f=0.01 is constructed, where the maximum hyperdegree of the network is 17. Finally, a directed social hypernetwork with the scale of N=5000, $n_{0}=6$, $n_{1}=2$, $n_{2}=5$ and ε=0.8 is generated, with its maximum in-degree reaching 866. The other simulation parameters are set as follows: α=0.3, β=0.6, η=0.2, γ=0.2. Figure 10 shows the propagation curves of node densities in various states with time evolution under the three different network structures.

It can be observed from Figure 10 that in Figure 10A BA scale-free hypernetwork and Figure 10B NW small-world hypernetwork, the proportion of I-state nodes both shows a trend of rising rapidly to a peak and then declining slowly. In contrast, in Figure 10C directed social hypernetwork, the proportion of I-state nodes presents a gentle evolutionary process of rising slowly to a peak and then declining slowly, with the propagation peak being significantly lower. This difference stems from the essential differences in the topological structures of the three types of networks. In the directed social hypernetwork constructed in this paper, information flows strictly along the propagation paths specified by directed hyperedges, and its directionality constraints inhibit the disorderly diffusion of information, thus reducing the propagation speed and narrowing the diffusion range. In comparison, the BA scale-free hypernetwork relies on a small number of highly connected hub nodes to rapidly promote information diffusion in the early stage of propagation, forming a higher propagation peak and a wider coverage range with a significantly faster propagation speed. The NW small-world hypernetwork has both a shorter average path length and a higher clustering coefficient, leading to high connectivity efficiency between nodes. Its propagation speed and range are both superior to those of the directed hypernetwork. However, the node degree distribution of the NW small-world hypernetwork is relatively uniform, and it mainly relies on local propagation mechanisms, resulting in an overall diffusion capacity still weaker than that of the BA scale-free hypernetwork.

In real social networks, information propagation exhibits the characteristics of strong suddenness, rapid fading, and short life cycle [32]. In the initial stage of information release, most users are in the latent state. With the enhancement of platform recommendation and user interaction, the number of directed hyperedges increases, and the information reachable range expands rapidly. When the social influence received by a node through directed hyperedges exceeds the propagation threshold, its state transforms from the latent state to the infectious state, thus driving the discussion popularity to rise rapidly in a short time. Subsequently, affected by frequent content updates and attention transfer, infectious nodes gradually transform into the recovered state, the number of effective propagation paths formed by directed hyperedges decreases, and the information diffusion capacity declines rapidly, forming a typical “outbreak-fading” propagation process. The above analysis indicates that the directed hypernetwork propagation model proposed in this paper presents certain advantages in characterizing the dynamic characteristics of information propagation on social platforms. Therefore, the subsequent simulation experiments will be carried out based on this model to further verify its applicability and effectiveness.

### 5.4. Impact of the Number of Followed Nodes on Information Propagation

**Figure 11:**
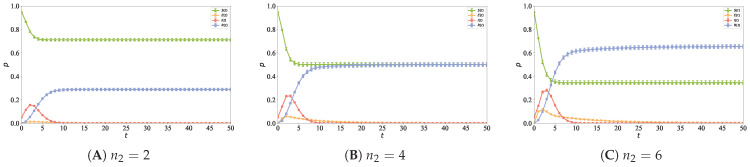
Different numbers of followed nodes ($n_{2}$) result in distinct information propagation curves, with (**A**) representing $n_{2}=2$, (**B**) $n_{2}=4$, and (**C**) $n_{2}=6$, respectively.

To investigate the impact of the number of followed nodes at each time step on the information propagation process, the number of followed nodes $n_{2}$ at each time step is set to 2, 4, and 6, respectively. The other simulation parameters are set as follows: N=5000, $n_{0}=6$, $n_{1}=2$, ε=0.8, α=1, ρ=0.05, coi=0.2, and λ=0.1. Figure 11 shows the information propagation curves under different values of the number of followed nodes $n_{2}$ in the directed social hypernetwork, which is used to explore the impact of enhanced network connectivity on the information diffusion process and propagation effect, where Figure 11A is for $n_{2}=2$, Figure 11B for $n_{2}=4$, and Figure 11C for $n_{2}=6$.

It can be observed from Figure 11 that with the increase in $n_{2}$ (the number of connected old nodes at each time step), the proportion of susceptible nodes in the steady state stage decreases gradually, the density peaks of exposed and infectious nodes increase continuously, and the final proportion of recovered nodes also increases accordingly. The results show that a larger $n_{2}$ enhances the connectivity and information accessibility of the network, enabling nodes to contact more information sources simultaneously in the initial stage, thus increasing the probability of being influenced. Under the directed hypernetwork structure, the expansion of connection scale further strengthens the information aggregation effect in high-order interactions, making it easier for information to reach the propagation threshold and trigger node state transition, thereby enhancing the overall information diffusion process.

### 5.5. Influence of the In-Degree and Out-Degree Weight Parameter ε on Information Propagation

**Figure 12:**
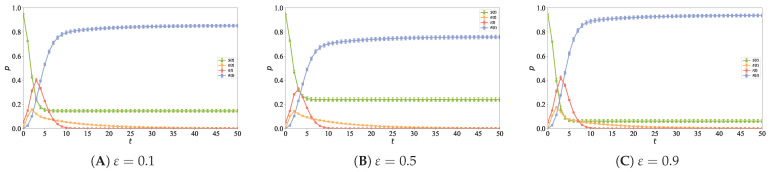
Information propagation curves under different weights, where panel (**A**) corresponds to ε=0.1, panel (**B**) to ε=0.5, and panel (**C**) to ε=0.9.

To investigate the impact of different ε values on the information propagation process in the directed hypernetwork, ε was set to 0.1, 0.5, and 0.9, while other simulation parameters were set as N=5000, $n_{0}=6$, $n_{1}=2$, $n_{2}=5$, α=1, ρ=0.05, and λ=0.1. Figure 12 shows the evolution curves of node densities in each state over time for different ε values, which are used to analyze the effect of in-degree and out-degree weights on the directed hypernetwork structure and the propagation process.

As shown in Figure 12, in the preferential attachment-based directed social hypernetwork, the scale of information propagation varies with the weight parameter ε in a pattern of first decreasing and then increasing, reaching its minimum at ε=0.5. When ε is biased toward in-degree (ε=0.9), new nodes tend to connect to high-follower core nodes, forming a network structure dominated by many-to-one B-hyperedges, which endows the core nodes with strong convergence and diffusion capabilities. When ε is biased toward out-degree (ε=0.1), the network concentrates on highly active nodes, increasing the proportion of one-to-many F-hyperedges, enabling super-active nodes to efficiently propagate information through multiple outgoing hyperedges. When ε balances in-degree and out-degree (ε=0.5), node connections are more uniform, structural heterogeneity is reduced, and the network lacks core hub nodes, resulting in slower information diffusion along directed hyperedges and the lowest steady state infection density. These results indicate that extreme preferential biases can enhance network heterogeneity and improve information propagation efficiency, while intermediate weights weaken the preferential effect and suppress large-scale diffusion. In addition, compared with out-degree, in-degree plays a more critical role in forming propagation cores and enhancing information convergence.

### 5.6. Impact of User Confidence on Information Propagation

**Figure 13:**
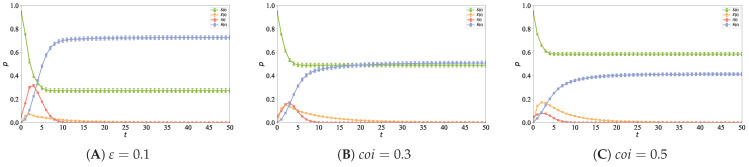
Different mean values of user confidence (coi) lead to distinct information propagation curves, with (**A**) for coi=0.1, (**B**) for coi=0.3, and (**C**) for coi=0.5.

To investigate the impact of threshold settings under different confidence mappings on the information propagation process in directed hypernetworks, the mean values of user confidence are set to 0.1, 0.3, and 0.5, respectively. The other simulation parameters are set as follows: N=5000, $n_{0}=6$, $n_{1}=2$, ε=0.8, α=1, ρ=0.05, and λ=0.1. Figure 13 shows the propagation curves of node densities in various states with time evolution under different mean values of user confidence in the hypernetwork, which is used to explore the differences in information propagation intensity, propagation speed, and steady-state distribution under different confidence settings, where Figure 13A is for coi=0.1, Figure 13B for coi=0.3, and Figure 13C for coi=0.5.

It can be observed from Figure 13 that with the increase in the mean value of high-confidence nodes in the network, the proportion of susceptible nodes increases gradually, the density peak of infectious nodes decreases gradually, and the density of recovered nodes in the steady state decreases gradually. The results show that users with high confidence have stronger rationality and prudence in information judgment. When exposed to new information, they are not immediately activated as propagators, but require cumulative influence from more in-neighbors before state transition occurs. This inhibits the propagation of low-quality information in the network, leading to a slower overall propagation speed and a narrower propagation range.

### 5.7. Impact of Information Timeliness on Information Propagation

**Figure 14:**
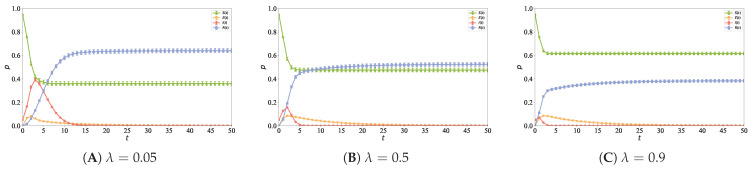
This figure shows the impact of the information timeliness characteristic λ on information propagation, where (**A**) corresponds to λ=0.05, (**B**) to λ=0.5, and (**C**) to λ=0.9.

To further investigate the impact of information timeliness on the information propagation process, the network information timeliness characteristic λ is set to 0.05, 0.5, and 0.9, respectively. The other simulation parameters are set as follows: N=5000, $n_{0}=6$, $n_{1}=2$, $n_{2}=5$, ε=0.8, α=1, ρ=0.05, and coi=0.2. Figure 14 shows the impact of information timeliness on information propagation, which is used to explore the effects of changes in the timeliness parameter on propagation speed, propagation peak and final steady state distribution, where Figure 14A is for λ=0.05, Figure 14B for λ=0.5, and Figure 14C for λ=0.9.

It can be observed from Figure 14 that with the increase in the timeliness parameter λ, the density peak of I-state nodes decreases gradually and approaches zero in the steady state, the density of R-state nodes in the steady state decreases synchronously, and the time required for the network to reach the steady state is also shortened gradually. The results show that the increase in the timeliness parameter λ shortens the effective survival time of information in the network, namely, the life cycle of information propagation is shortened. With the accelerated attenuation of information influence, it is more difficult for nodes to maintain the propagation state for a long time, and propagation nodes turn from the active state to the recovered state faster, thus interrupting the propagation chain in advance and limiting the information diffusion range. Therefore, with the increase in λ, the peak of information propagation decreases, the overall scale shrinks, and the system enters a steady state faster.

### 5.8. Comparison with Information Propagation in Undirected Hypernetworks

**Figure 15:**
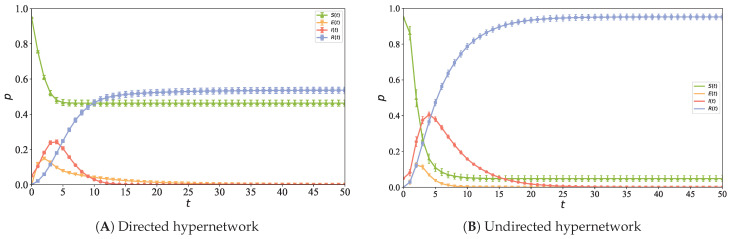
Information propagation curves vary across different hypernetwork structures with (**A**) corresponding to the directed hypernetwork and (**B**) corresponding to the undirected hypernetwork.

To compare the differences in the information propagation process between undirected and directed hypernetwork structures, an undirected hypernetwork with a node scale of N=5000 is constructed according to the method in [33], and the initial infected node proportion is set to ρ=0.05. The evolution process of node densities in different states with time is then simulated in this network structure, and the propagation curves are shown in Figure 15, where Figure 15A shows the directed hypernetwork and Figure 15B shows the undirected hypernetwork. This figure is used to analyze the impact of network directionality on propagation speed, propagation range, and steady state distribution.

Figure 15 shows the change curves of different node densities with time during the information propagation process in the directed and undirected hypernetworks, respectively. In Figure 15A, due to the directionality constraint on information propagation, the propagation paths are relatively limited, resulting in a lower peak of the infectious node density curve and a smaller propagation range. With the increase in time steps, the density curve of susceptible nodes gradually decreases and tends to be stable; the density curve of exposed nodes rises slowly to a peak in the early stage and then decreases slowly; the density curve of infectious nodes reaches a peak at the fourth time step and then decreases; finally, most nodes enter the recovered state, and the density curve of recovered nodes tends to be steady after the 27th time step. In contrast, in Figure 15B, due to the bidirectional influence between nodes, the propagation chain is longer and the diffusion is more sufficient, resulting in a higher peak of the infectious node density curve than that of the directed hypernetwork and a larger propagation scale; the density curve of susceptible nodes decreases rapidly, and the density curve of exposed nodes is almost exhausted in a short time; finally, the density curve of recovered nodes enters the steady state earlier than that of the directed hypernetwork.

Significant differences are observed in the information propagation dynamics between directed and undirected hypernetworks: directionality constraints weaken the propagation efficiency, while bidirectional interactions significantly enhance the diffusion capacity. In directed hypernetworks, unidirectional information flow limits the reachable paths, resulting in a lower peak of infectious node density, a limited propagation range, and a slower and incomplete overall propagation process. On the contrary, in undirected hypernetworks, the bidirectional reachability of undirected connections enables information to cover a wider range of nodes, significantly improving the propagation speed and scale, leading to a higher peak of infectious node density, a faster exhaustion of susceptible nodes, and an earlier steady state of recovered nodes. The comparison shows that the existence of hyperedge directionality is an important structural factor determining the intensity of group propagation and the depth of diffusion. In real social scenarios, information propagation is mainly carried out through follow relationships between users, and information is usually diffused unidirectionally from followed users to followers along follow links. This directional high-order propagation process dominated by follow relationships is difficult to characterize by traditional undirected hypernetworks. Therefore, the information propagation model based on directed hypernetworks can more truly reflect the information diffusion mechanism on social platforms.

### 5.9. Comparison of Propagation with Classical Infectious Disease Models

To verify the advantages of the proposed directed hypernetwork propagation model in characterizing information propagation characteristics, this section conducts a comparative analysis between it and the classical infectious disease models SIR and SEIR. By comparing the performance of different models in terms of propagation speed, propagation scale, and steady state node distribution, the influences of directed high-order structures and node behavioral characteristics on the propagation process are deeply understood, and the improvement effect of the constructed model compared with traditional network models is evaluated.

**Figure 16:**
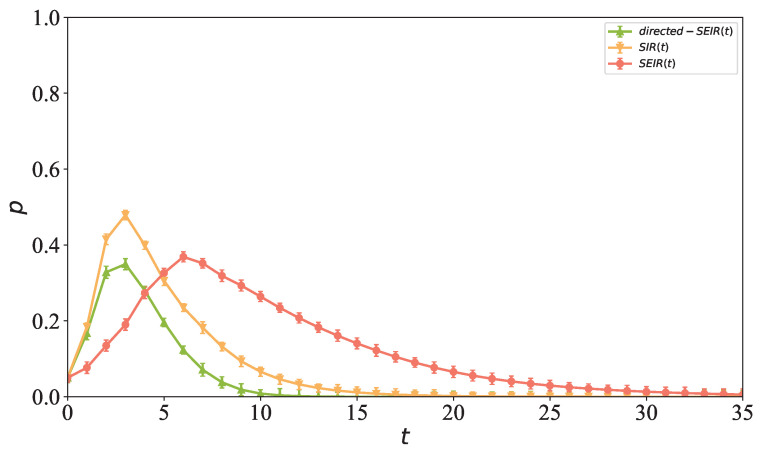
Comparison between the proposed directed hypernetwork propagation model and classical propagation models. The yellow curve, red curve, and green curve represent the evolution curves of the density of infected nodes over time in the classical SIR model, classical SEIR model, and the proposed directed hypernetwork propagation model, respectively.

It can be seen from Figure 16 that the directed hypernetwork propagation model proposed in this paper exhibits significant differences from the classical SIR and SEIR propagation models in terms of propagation speed, peak scale, and duration. The density of infected nodes in the classical SIR model rises the fastest and reaches the peak at the third time step, showing a typical rapid outbreak characteristic. Due to the existence of the latent period, the propagation process of the classical SEIR model has an obvious lag, reaching the peak at the seventh time step with the slowest decay process. In contrast, the density of infected nodes in the proposed model also reaches the peak at the third time step, but the peak level is lower than those of the classical SIR and SEIR models, and tends to be steady after the 17th time step with an earlier termination of the propagation process. The results indicate that, under the same network structure, the proposed model not only retains certain propagation capability, but also effectively suppresses the concentrated outbreak of infected nodes and shortens the propagation duration. This is mainly because the proposed model considers not only the transition process of propagation states, but also the constraints of information propagation under directed high-order interaction characteristics, enabling the propagation process to better reflect the evolutionary characteristics of information diffusion in social networks affected jointly by directional relationships and group effects. From the epidemiological perspective, this feature means that the model can simulate the high-order contact effects and propagation restriction phenomena of real disease transmission, i.e., a single infection source no longer triggers a global outbreak, but is constrained by the group structure and directional transmission chains, thus being closer to the development law of real infectious diseases. Compared with the classical SIR/SEIR models, the proposed model presents better authenticity and explanatory power in characterizing the peak delay, propagation intensity control and propagation life cycle shortening of disease transmission, and also provides a feasible modeling framework for the study of comorbidities and complex transmission paths.

## 6. Conclusions and Discussion

In this paper, a directed hypernetwork propagation model based on the BA scale-free network is proposed to characterize the group attention structure and information propagation behavior in social platforms. The model characterizes the directionality and functional differences of social relationships through different types of directed hyperedges, reflects the characteristics of information flow and group interaction in the structures, and provides a structured framework for analyzing the organization of information diffusion in social platforms. On this basis, combined with a threshold mechanism based on user confidence, the state transition of nodes is made more consistent with the logic of information selection and propagation in actual platforms. Taking the “imposition of tariff event” as an example, the hyperdegree distribution of the directed hypernetwork generated by the model is compared with real data. The results show that both exhibit a power-law distribution with typical scale-free characteristics. Further comparative analysis between the real propagation trend and the model simulation results reveals that the two maintain high consistency in the propagation trend and peak position, verifying the rationality and effectiveness of the model. In addition, experimental verification of the topological structure and propagation process of the model was conducted based on seven public datasets. The results show that the proposed model can accurately reflect the structural characteristics and propagation dynamics of the network, and has strong applicability and robustness. Simulation results show that there are significant differences in information propagation effects under different network structure conditions, and the directional constraints in directed hypernetworks have an important impact on the propagation process. With the increase in the number of nodes followed by newly added nodes at each time step, the network connectivity and information reachability are enhanced. Meanwhile, a higher propagation threshold can enhance users’ information screening ability and significantly inhibit the diffusion of low-quality information. After removing the directional constraints, the information propagation speed is accelerated and the coverage is expanded. This study provides a theoretical basis for public opinion monitoring, rumor governance, and the design of propagation intervention strategies.

Although the proposed model can comprehensively capture the main propagation characteristics on social platforms, there is still room for further expansion. For example, the multi-level feedback mechanism of recommendation algorithms, the dynamic evolution of user interests, and the differentiated propagation mechanisms of different content types have not been incorporated into the model. Future research can integrate processes such as platform push mechanisms, user emotional factors, and multi-topic competition into the model. Meanwhile, systematic verification of the model with more real social platform data will help us to further improve the explanatory and predictive power of the model, thereby enhancing its applicability in practical application scenarios such as public opinion management and propagation intervention.

## Figures and Tables

**Table 1:** Topological structures of datasets.

	Metabolic		Citation		Q&A		Bitcoin			Directed Social Hypernetwork
	iAF1260b	iJO1366	Data Mining	Software	Math	Server	2014	2015	2016	
Total Nodes	1668	1805	27,164	16,555	34,812	172,330	1,697,625	1,961,886	2,009,978	5000
Total Hyperedges	2083	2251	73,113	53,177	93,731	272,116	1,437,082	1,449,827	1,451,135	6050
$d_{\max}^{\mathrm{in}}$	701	751	2520	941	888	360	49,586	26,480	10,903	1235
$d_{\min}^{\mathrm{in}}$	1	1	1	1	1	1	1	1	1	1
$d_{\mathrm{avg}}^{\mathrm{in}}$	3.60	3.54	13.29	13.17	3.57	2.28	1.59	1.48	1.43	17.629
$d_{\max}^{\mathrm{out}}$	425	457	799	433	1946	5071	45,526	24,729	13,078	5
$d_{\min}^{\mathrm{out}}$	1	1	1	1	1	1	1	1	1	1
$d_{\mathrm{avg}}^{\mathrm{out}}$	2.72	2.75	9.87	10.65	10.54	4.34	1.65	1.48	1.49	1.421
$\gamma_{\mathrm{in}}$	2.048	2.052	2.063	1.878	1.948	2.505	2.279	2.288	2.403	1.392
$\gamma_{\mathrm{out}}$	1.909	1.875	2.256	2.332	1.675	1.902	2.236	2.092	2.522	1.475
$\gamma_{\mathrm{total}}$	2.321	2.336	2.266	2.096	1.777	2.098	2.331	2.355	2.508	1.479

## Data Availability

The data presented in this study are available on request from the corresponding author. The data are not publicly available due to privacy concerns for the participants involved in the study.
